# Supplementary material for: Mdm2/p53 levels in bone marrow mesenchymal stromal cells are essential for maintaining the hematopoietic niche in response to DNA damage
Source: Cell Death Dis. 2023 Jun 23;14(6):371. doi: 10.1038/s41419-023-05844-7 (PMC10290070; doi:10.1038/s41419-023-05844-7)
Supplement: Supplementary file 2 — Supplementary Figure Legends [file 41419_2023_5844_MOESM2_ESM.docx]

**Supplementary Figure S1: *Osx-Cre* marks MSCs in neonatal bones and adult MSCs** (A and B) A direct fluorescence image of bone sections of a 2-month old *Osx-Cre;mTmG* mouse showing the distribution of GFP+ cells in the bone marrow. A closer view of the growth plate is marked by the red box. Immunofluorescence staining of perilipin (magenta) and leptin receptor (LepR) as indicated. Yellow arrows mark MSCs. (C) Flow cytometric analysis of MSCs derived from *Osx-mTmG* mouse using the indicated antibodies. D) Hematological analysis of *Osx-Mdm2^+/+^* and *Osx-Mdm2^fl/+^* mice. Data points represent individual mice, and bars indicate the mean ± standard deviation (SD). The graph shows the total white blood cell (WBC), platelet (PLT), and red blood cell (RBC) counts in both groups. Student's t-test was used to compare the means, ns; not significant. E) A direct fluorescence image of a fetal skull from an *Osx-Cre;mTmG* mouse showing GFP expression in developing bone is presented. The brain, which is devoid of GFP, is also visible in the image. White arrows are used to indicate the skull bone. In the right panel, a section of the liver is shown, demonstrating no expression of GFP.

**Supplementary Figure S2: Deletion of p53 in MSCs does not impact trabecular bone density** (A) Representative flow cytometry plots and quantification of the expression of lineage markers CD150, c-Kit, and Sca-1 in the indicated mice (mean ± SD). *P* = 0.88, n = 3. (B) Quantification of trabecular bone mean density, trabecular bone volume, and bone volume relative to total volume in *Osx-Cre;Trp53^Wt^* and *Osx-Cre;Trp53^fl/fl^* (n = 6, ns: not significant). (C) Representative micro-CT scans of the indicated mice. The white arrows mark the trabecular bone (D) Immune staining of GFP in a bone section derived from *Osx-Cre;Trp53^fl/fl^;Mdm2^fl/+^;mTmG* mice. The red area is magnified on the adjacent image. The red arrows mark GFP+ MSCs.

**Supplementary Figure S3:** Cytof imaging was performed on frozen bone marrow sections from *Osx-cre;Trp53^fl/fl^; mTmG* and *Osx-cre;Trp53^+/+^; mTmG* transplanted with AML cells and treated with DS5272. The panel A shows the distribution of AML cells (CD44+), MSCs (GFP+), erythroid cells (CD71+Ter119+) in the bone marrow. B) The average expression levels of each marker (y axis) in different phenotypic clusters (x axis) were determined using the MCD viewer and plotted as a violin plot. Three different regions of interest (ROI) were analyzed, and marker expression data for each cell were calculated as the mean intensities of ion counts over the object mask. C) Immunoflourecence image of Edu staining in the femur of indicated mice. The panel on the right shows a high magnification of the merged signals of Dapi, AML cells, and Edu. D) Quantification of Edu-expressing cells in each group by normalizing the number of Edu-expressing cells to Dapi. Each data point represents the ratio of Edu+ cells to Dapi in one high-power field (HPF) and is presented as the mean ± SD, Student’s t test, ***P* < 0.01.
