# Supplementary figures and images for: Mdm2/p53 levels in bone marrow mesenchymal stromal cells are essential for maintaining the hematopoietic niche in response to DNA damage
Source: Cell Death Dis. 2023 Jun 23;14(6):371. doi: 10.1038/s41419-023-05844-7 (PMC10290070; doi:10.1038/s41419-023-05844-7)

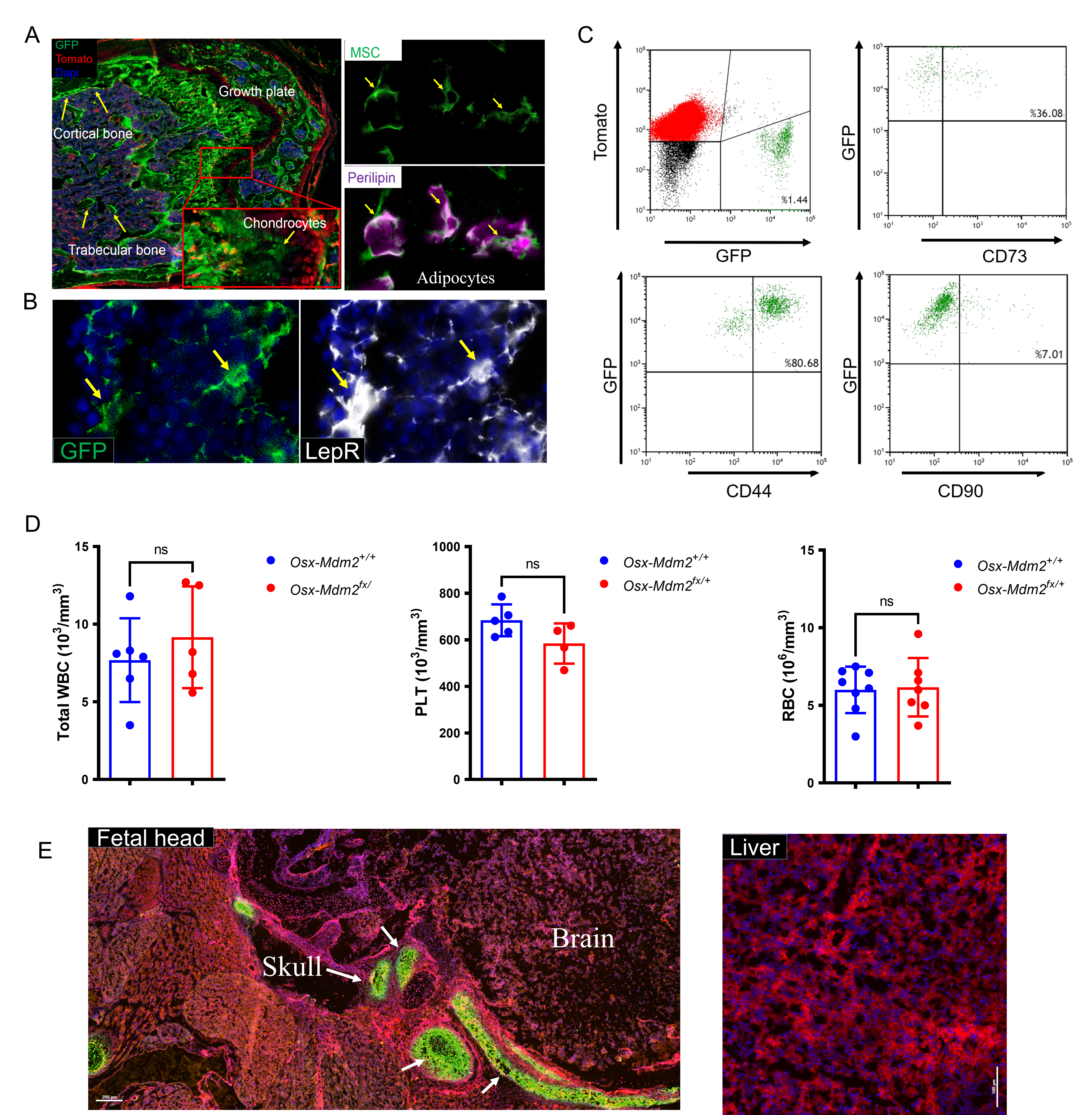

Supplement: Supplementary file 3 — Supplementary Figure 1 [file 41419_2023_5844_MOESM3_ESM.tif]

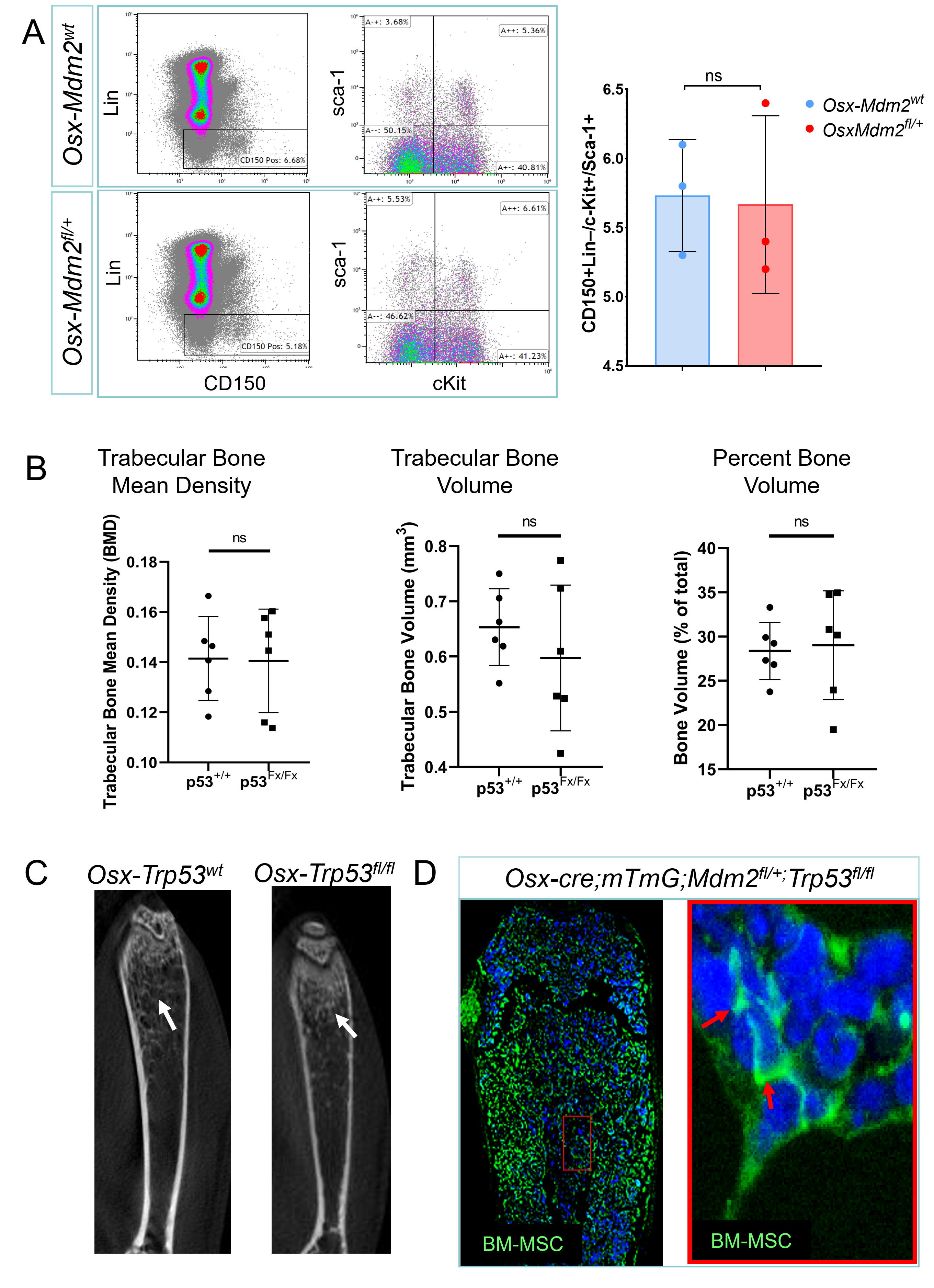

Supplement: Supplementary file 4 — Supplementary Figure 2 [file 41419_2023_5844_MOESM4_ESM.tif]

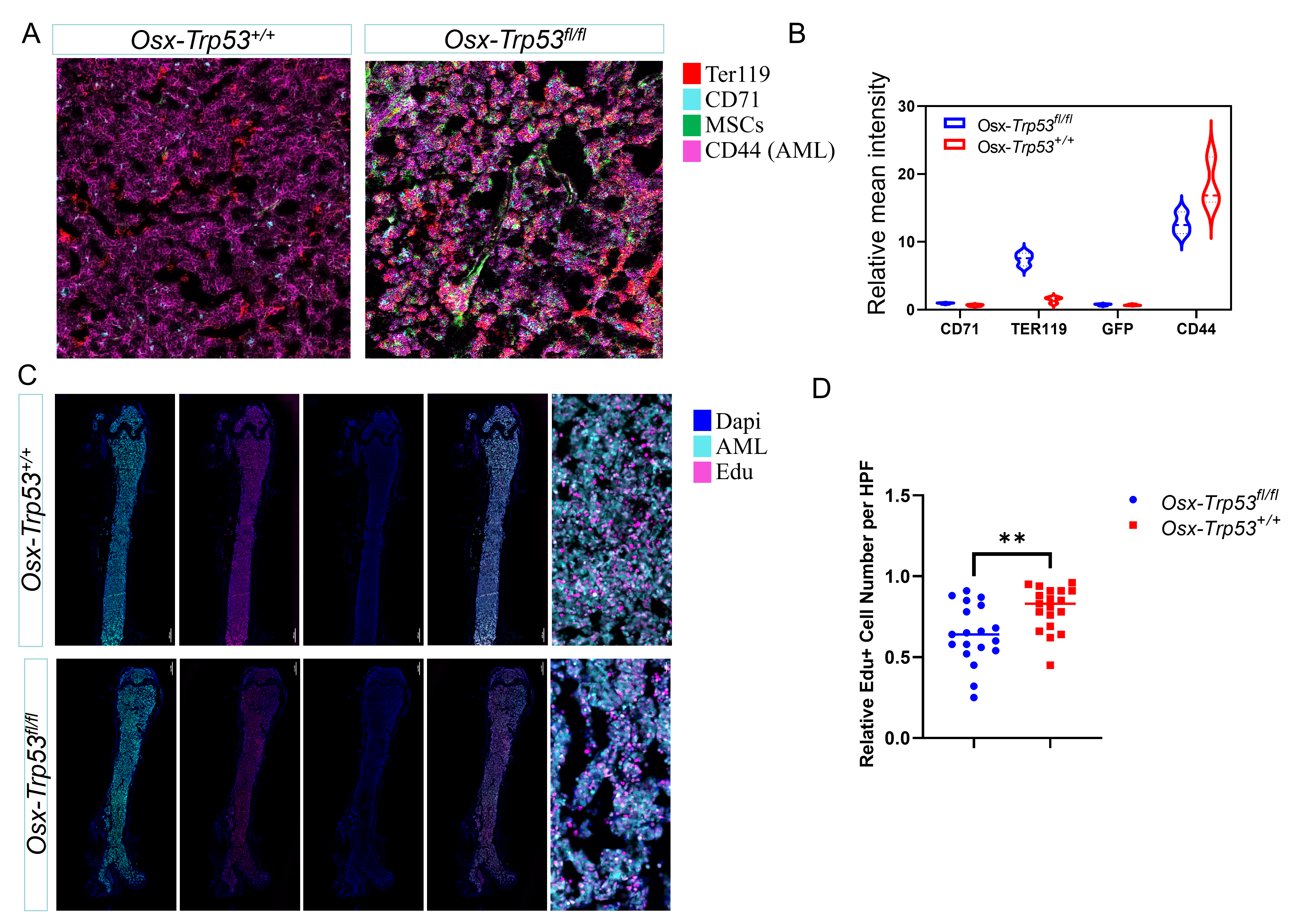

Supplement: Supplementary file 5 — Supplementary Figure 3 [file 41419_2023_5844_MOESM5_ESM.tif]
